# Influence of Moisture Content and Wet Environment on the Fatigue Behaviour of High-Strength Concrete

**DOI:** 10.3390/ma15031025

**Published:** 2022-01-28

**Authors:** Mohamed Abubakar Ali, Christoph Tomann, Fadi Aldakheel, Markus Mahlbacher, Nima Noii, Nadja Oneschkow, Karl-Heinz Drake, Ludger Lohaus, Peter Wriggers, Michael Haist

**Affiliations:** 1 Institute of Building Materials Science, Leibniz University Hannover, Appelstraße 9a, 30167 Hannover, Germany; m.mahlbacher@baustoff.uni-hannover.de (M.M.); n.oneschkow@baustoff.uni-hannover.de (N.O.); k-h.drake@baustoff.uni-hannover.de (K.-H.D.); lohaus@baustoff.uni-hannover.de (L.L.); haist@baustoff.uni-hannover.de (M.H.); 2 LPI Ingenieurgesellschaft, Völgerstraße 9, 30519 Hannover, Germany; tomann@lpi-ing.de; 3 Institute of Continuum Mechanics, Leibniz University Hannover, an der Universität 1, 30823 Garbsen, Germany; aldakheel@ikm.uni-hannover.de (F.A.); noii@ikm.uni-hannover.de (N.N.); wriggers@ikm.uni-hannover.de (P.W.); 4 Zienkiewicz Centre for Computational Engineering, Faculty of Science and Engineering, Bay Campus, Swansea University, Swansea SA1 8EN, UK

**Keywords:** high-strength concrete, moisture content, fatigue deterioration, water-induced degradation mechanisms, acoustic emissions analysis, phase-field approach, porous media theory, microscopic material model

## Abstract

The influence of a wet environment on the fatigue behaviour of high-strength concrete has become more important in recent years with the expansion of offshore wind energy systems. According to the few investigations documented in the literature, the fatigue resistance of specimens submerged in water is significantly lower compared to that of specimens in dry conditions. However, it is still not clear how the wet environment and the moisture content in concrete influence its fatigue behaviour and which damage mechanisms are involved in the deterioration process. Here the results of a joint project are reported, in which the impact of moisture content in concrete on fatigue deterioration are investigated experimentally and numerically. Aside from the number of cycles to failure, the development of stiffness and acoustic emission (AE) hits are analysed as damage inductors and discussed along with results of microstructural investigations to provide insights into the degradation mechanisms. Subsequently, an efficient numeric modelling approach to water-induced fatigue damage is presented. The results of the fatigue tests show an accelerated degradation behaviour with increasing moisture content of the concrete. Further, it was found that the AE hits of specimens submerged in water occur exclusively close to the minimum stress level in contrast to specimens subjected to dry conditions, which means that additional damage mechanisms are acting with increasing moisture content in the concrete.

## 1. Introduction

The number of fatigue-loaded concrete structures in wet environments has significantly increased in recent years with the expansion of offshore wind energy systems. Such structures are exposed to millions of load cycles within their service life due to wind, waves and rotor movement. In contrast to dry fatigue-loaded structures, a significantly higher water content in the microstructure of the concrete occurs as a result of the offshore exposure. Fatigue investigations documented in the literature indicate a significantly lower fatigue resistance of high-strength concrete specimens submerged in water compared to those exposed to dry conditions [1,2,3,4,5,6,7]. Furthermore, concrete specimens in wet environments, i.e., specimens stored and tested in water, show a different fracture behaviour in contrast to specimens in dry environments. This is indicated during testing by ascending air bubbles, the washout of fine particles into the surroundings and premature crack initiation [6]. *Oneschkow* et al. [4] found indications that the influence of the loading frequency is higher for specimens stored and tested in a wet environment compared to those subjected to dry conditions. In addition to the numbers of cycles to failure, the specific shape of the well-known S-shaped strain and stiffness development curves with phases I–III is often used in the literature as an indicator of the fatigue damage of concrete (see, e.g., [2,5,8]). According to *Hümme* [5], a higher stiffness degradation per load cycle can be observed for specimens in wet environments compared to dry specimens.

Although the phenomenon of the water-induced damage of cyclically loaded concrete has been recognised in the literature, there is still a pronounced lack of knowledge concerning the underlying damage mechanisms involved in the deterioration process. Various hypotheses regarding water-induced damage mechanisms have been documented in the literature, but these have not yet been experimentally validated (see, e.g., [3,5,9,10,11]). According to *Sørensen* [3] and *Hümme* [5], for example, pressure can build up within water-saturated pores due to fatigue loading. At a critical limit, this pressure induces an additional local tensile stress in the microstructure of the concrete, which leads to an accelerated fatigue damage progress. Another explanation points to possible water transport within the pore system of the cement paste due to fatigue loading [11]. Numerical modelling and simulation of damage mechanisms due to the impact of water are not yet available because of the existing knowledge gap. This was the motivation to investigate and develop a new multi-physical model to simulate these mechanisms, as documented in [12].

Well-instrumented fatigue investigations on high-strength concretes with varied moisture contents have been conducted in a joint project of the Priority Programme SPP 2020 ‘Cyclic Deterioration of High-Performance Concrete in an Experimental-Virtual Lab’, funded by the German Research Foundation. The main aim of the project was to investigate and describe the water-induced damage mechanisms of fatigue loaded high-strength concretes. A numerical model for water-induced damage mechanisms has been developed and implemented additionally to assess the hypotheses derived from the experimental fatigue investigations. The model is validated in further stages of the project from the results of microstructural investigations such as nuclear magnetic resonance (NMR) or nano-indentation testing. Moreover, the influence of load frequencies on the water-induced fatigue damage was investigated in another project.

In this paper, the respective number of cycles to failure of fatigue loaded high-strength concretes with varied moisture contents are presented. The development of stiffness and acoustic emission (AE) hits are additionally analysed as damage indicators and discussed along with the results of microstructural investigations to provide insight into the moisture-dependent degradation mechanisms. A numerically phenomenological description of fatigue damage is presented by combining the phase-field approach to damage with a porous media theory. A local lifetime variable is obtained that reflects the progressive degradation of fatigue damage resistance.

## 2. Materials and Methods

### 2.1. Concrete Composition and Specimen Preparation

The first part of the fatigue tests was carried out on a reference dry mix of high-strength concrete in the Priority Programme SPP 2020 called RH1 [13,14]. The concrete composition is detailed in Table 1. The reference concrete has a water to cement (w/c) ratio of 0.35 and the basalt aggregates have a maximum grain size of 8 mm. The compressive strength of the concrete at age of 28 days was tested according to [15] by using 150 mm cubic samples after underwater storage. The 28 days mean compressive strength was determined as *f_cm_*_,150_ = 113 MPa. The second part of the investigation considering various load frequencies was carried out on a dry mix of high-strength concrete for which the detailed composition was not provided. The concrete used was assigned to the strength class C90/105, according to the manufacturer’s specifications and called HPC-1 in following.

Cylindrical specimens with a height of *h* = 300 mm and diameter of *d* = 100 mm were used in the investigations. Regarding the manufacturing process, the concrete was filled into plastic formworks in two equal layers and each layer compacted on a vibratory table. The formworks were removed two days after concreting. The test specimens were cut at both ends and prepared by grinding and fine polishing to ensure a centric loading and a uniform stress distribution. The different moisture contents of the concrete were adjusted as outlined below. Table 2 contains an overview of the storage and test conditions.

Test specimens in **series D** were dried at 105 ± 5 °C to a constant mass, beginning at an age of 90 days. These specimens only contain non-evaporable, essentially chemically bound water in their microstructure. Specimens in **series C** were stored in a climate chamber under standardized conditions (20 °C, 65% RH) for at least 90 days, beginning immediately after the concreting process. Moisture gradients are reduced and the specimens show a lower overall moisture content due to the long storage time. Direct moisture exchange between the test specimens and the environment was prevented in **series M** by sealing the specimens with aluminium-coated butyl tape immediately after the grinding process. **Series WS** consists of test specimens that were permanently submerged in water (directly after the concreting process until testing) and stored at a constant temperature of 20 °C. The test specimens in **series D**, **C** and **WS** were sealed before testing to prevent drying during the fatigue tests. The specimens in the **series** **WST** were stored in a manner similar to the **WS series**. However, these specimens were tested underwater without being sealed. Fatigue tests were carried out on concrete specimens with a minimum age of 90 days.

Microstructural investigations were carried out on mortar samples. Fresh concrete of the RH1 mix was sieved to 2 mm to retain the typical concrete matrix composition. This mortar was cast in tubes with a diameter of 10 mm, moisture-sealed during curing, cut to 30 mm lengths after 28 days and polished at the end faces. The specimens were stored for over 90 days submerged in water to receive moisture conditioning of type **WS**, i.e., full saturation. One week prior to the fatigue exposure, the specimens were moisture-sealed with epoxy resin coating (except for the end faces) to prevent the samples from drying during testing.

### 2.2. Test Programme and Experimental Set-Up

**Figure 1:**
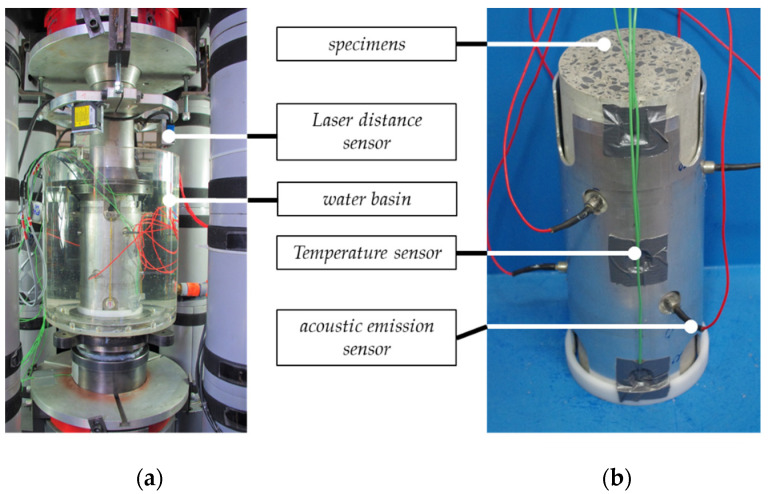
Experimental set-up (**a**) and measurement equipment (**b**).

The macroscopic experimental investigations were performed in a 2.5 MN class 0.5 servohydraulic testing machine (according to ISO 7500-1 [16]). A specially developed cylindrical water basin was used (Figure 1a) for the fatigue tests on concrete specimens submerged in water. The water basin set-up enables experiments with specimens which are fully submerged in water. The cyclical tests were carried out force–controlled with a sinusoidal load after a monotonic increase of force of 0.5 MPa/s until the mean stress level. The minimum and maximum stress levels, and load frequency were kept constant throughout the entire investigation period with *S_min_* = 0.05, *S_max_* = 0.65 and *f_t_* = 1.0 Hz. The reference compressive strength of the concrete was determined before the cyclical tests as the mean value of three compressive strengths by using the specimens from the same batch and storage conditions as in cyclical tests. An overview of the reference compressive strength for each concrete and the value of moisture content for each storage condition is given in Table A1 in Appendix A. Fatigue tests were conducted on prefabricated concrete specimens (HPC-1) from the **WST** series with different load frequencies of *f_t_* = 0.35, 1.0, 5.0 and 10.0 Hz to determine the impact of the load frequency on the water-induced fatigue deterioration. An overview of the number of macroscopic fatigue tests is given in Table 3.

The axial force, axial deformations, surface temperature and AE activity were measured during all cyclical tests with a sampling rate of 300 Hz. The axial deformations of the specimens were measured using three laser distance sensors positioned on the circumference, each offset by 120° to the other. Six AE sensors with a wideband frequency response within the range of 250 to 1600 kHz were attached to the specimens. The sensors were positioned at 60° from one another, alternately in the upper and lower third of the specimen. A specific threshold value of 40 dB was selected to separate useful AE signals (AE hits) from the background noise [12,17]. The measuring equipment used is illustrated in Figure 1b.

Microstructural investigations with the nuclear magnetic resonance (NMR) method were performed on moisture-sealed mortar samples from storage condition **WS**. The samples were wrapped in cling film to prevent drying during the NMR measurement and placed in a BRUKER minispec mq10 instrument (0.235 T) with a custom probe head. The T2 relaxation from the CPMG sequence was recorded for 1600 repetitions (for details regarding the measurement techniques, see [18]). After that, the specimens were placed in a 10 kN servohydraulic testing machine and exposed to a sinusoidal force-controlled load with a minimum stress level of *S_min_* = 0.05 and a maximum stress level of *S_max_* = 0.65 at a load frequency of *f_t_* = 1.0 Hz. Fatigue exposure was stopped manually at the end of phase II of observing strain recorded from the servo-hydraulic testing machine. The NMR testing was performed again within 1 h after stopping the fatigue exposure with the same settings. Data analysis was performed by multiexponential fitting of the relaxation signal with preselected T2-values similar to [19], to receive moisture quantities designated to different pore species. Analysis from inverse Laplace transformation using the algorithm described in [20] yielded three main relaxation components at T_2_ relaxation times of 0.1, 0.31 and 4.5 ms, respectively, owing to the small number of large porosities in typical HPCs. These would typically correspond to pore sizes of 1 nm, 2 to 5 nm and approx. 20 to 50 nm (compare [21]). Despite the very small nature of the pores detected, the pores are named “micro”, “meso” and “macro”, following the assignment in [22].

## 3. Results

### 3.1. Numbers of Cycles to Failure

**Figure 2:**
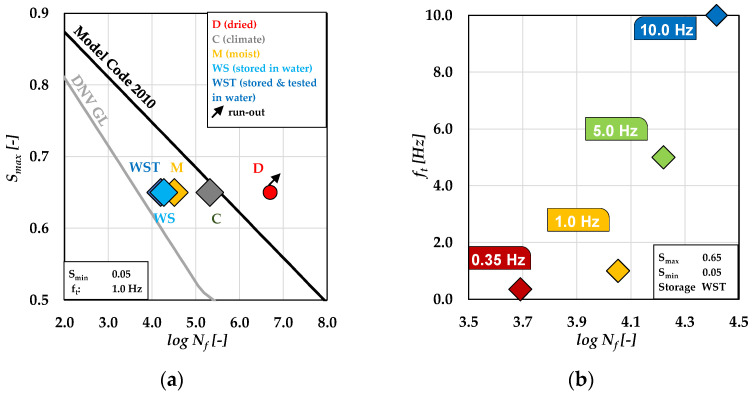
Numbers of cycles to failure depending on the storage condition (**a**) [22]; depending on the load frequency for storage condition **WST** (**b**).

A summary of the numbers of cycles to failure *N_f_* is presented as single and mean values in Table A2 and Table A3 in Appendix A. The mean values depending on the storage condition of the specimens are plotted in Figure 2a on a logarithmic scale (*log_10_ N_f_*). The S-N curves of DNV GL [23] for wet conditions and the S-N curve of *fib* Model Code 2010 [24] for dry conditions are included in the figure. The results show that the largest difference in the number of cycles to failure was found between the storage conditions **D** and **WST** (Δ*log_10_ N_f_* = 2.5). It should be mentioned that the result of the series **D** refers to one run-out, i.e., the real number of cycles to failure is unknown. The values of the storage conditions **C** and **M** are spread between the conditions **WST** and **D**, while the number of cycles to failure of the storage condition **M** is lower than for the condition **C**. The mean value of the storage condition **C** is located near to the value of *fib* Model Code 2010. It was found, additionally, that the results of numbers of cycles to failure of specimens in the series **WS** and **WST** are located close to each other. This indicates that the moisture content in the concrete—and not the wet environment—is substantially responsible for the accelerated fatigue damage. It is clear from Figure 2a that the number of cycles to failure decreases with an increasing moisture content in the concrete. This corresponds well to the results of the very limited number of investigations previously documented in the literature [1,2,3,4,5,6,7] and further ongoing research [25,26].

The numbers of cycles to failure depending on the load frequency are shown in Figure 2b on a logarithmic scale. Please note that the impact of the load frequency was investigated only for the storage condition **WST**. The impact of load frequency on fatigue behavior of high-strength concrete specimens with varied moisture contents are investigated and analysed by means of AE hits in further stages of the project. The test results for *f_t_* = 0.35 Hz show the lowest number of cycles to failure (*log_10_ N_f_* = 3.69). By contrast, specimens loaded with the highest load frequency of 10 Hz reach the highest number of cycles to failure (*log_10_ N_f_* = 4.42). Thus, the results suggest that the water-induced fatigue damage due to fatigue increases significantly with decreasing the load frequency.

### 3.2. Macroscopic Damage Indicators

The stiffness degradation per load cycle was analysed throughout the test duration depending on the moisture content of the concrete. The stiffness was calculated for each load cycle as the secant modulus (*E_s_*) in the decreasing branch of the sinusoidal strain curve using the strains at the maximum and minimum (peak) stresses. Based on the development of stiffness in the fatigue process, the gradient, i.e., the stiffness degradation per load cycle, in phase II of the development of stiffness is also analysed as damage indicator. The slope of the development of stiffness in phase II, referred to as $\mathrm{grad}\;E_{s}$, is calculated according to Oneschkow [27] between *N*/*N_f_* = 0.20 to 0.80 as follows:

(1) $${\mathrm{grad}\;E}_{s}^{0.2–0.8}=\left(\frac{\Delta E_{s}^{0.2–0.8}}{\Delta N^{0.2–0.8}}\right)$$

**Figure 3:**
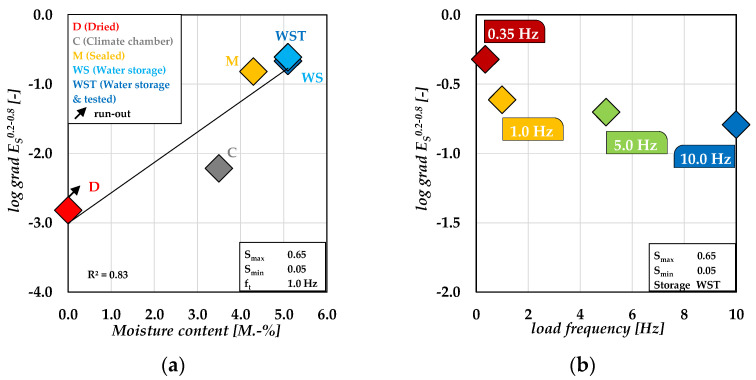
Logarithmic gradient of stiffness in phase II depending on the moisture content (**a**) [22] and depending on the load frequency for storage condition **WST** (**b**).

The results are shown as mean values in Figure 3a as function of the moisture content. The water-saturated specimens of the storage condition **WS** and **WST** show the highest stiffness reduction per load cycle. By contrast, specimens from storage condition **D** show the lowest value of logarithmic gradient of stiffness. The results from the storage conditions **M** and **C** are located in descending order between the water-saturated (**WST**) and dry specimen (**D**) with a rapidly decrease of the values from −2.23 to −0.82. The rapid decrease might indicate that additional damage mechanisms are acting when a certain value of moisture content in the concrete is exceeded. The mean values of the logarithmic gradient of stiffness in phase II depending on loading frequency are plotted in Figure 3b. It is obvious from the figure that specimens tested with the lowest load frequency of 0.35 Hz show the highest value of the gradient of stiffness. The lowest value was reached for the highest load frequency *f_t_* = 10.0 Hz.

In summary, the results of the logarithmic gradient of stiffness indicate that the value of stiffness reduction per load cycle increases with increased moisture content and decreased loading frequency. This correlates very well with the results of the number of cycles to failure.

**Figure 4:**
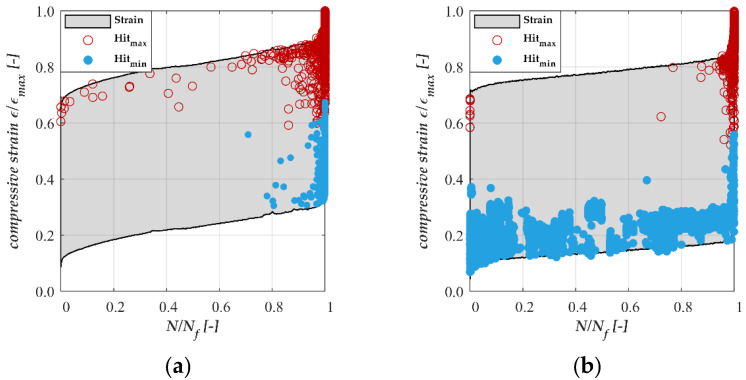
Representative acoustic emission signals during fatigue loading: (**a**) of storage condition **C**; (**b**) of storage condition **WS** [22].

The AE activity was analysed as a second damage indicator to obtain insight into the moisture-dependent fatigue deterioration of the concrete. Figure 4 illustrates the development of the AE activity throughout the test duration for one representative test specimen each for the storage conditions **C** (Figure 4a) and **WS** (Figure 4b), respectively. The grey shapes represent schematically the well-known S-shaped strain development curve. The red circles represent AE hits, which are oriented closer to the maximum stress level. These hits are called *Hit_max_* in the following. The blue points represent hits oriented closer to the minimum stress level and they are called *Hit_min_* in following. The results show considerable differences in AE activity between the two storage conditions **C** and **WS**. It can be seen from Figure 4a that the number of AE hits detected in phase I and in approximately two-thirds of phase II are smaller than the number of hits of the storage condition **WS** (Figure 4b). Those hits occur exclusively close to the maximum stress level (*Hit_max_*). However, the number of AE hits (*Hit_max_* and *Hit_min_*) increases rapidly in the last third of phases II and in phase III. By contrast, the AE hits of water-saturated test specimens occur mainly near to the minimum stress level (*Hit_min_*). Hits close to the maximum stress level can only be observed at the end of phase II and in phase III. In the latter phase III, the number of AE hits increases rapidly in a manner similar to storage condition **C**. The difference in the AE signals and signal occurrence between both storage conditions indicates that additional damage mechanisms are acting with increasing moisture content in the concrete, which led to an accelerated degradation process due fatigue loading.

### 3.3. Microstructural Investigations

**Figure 5:**
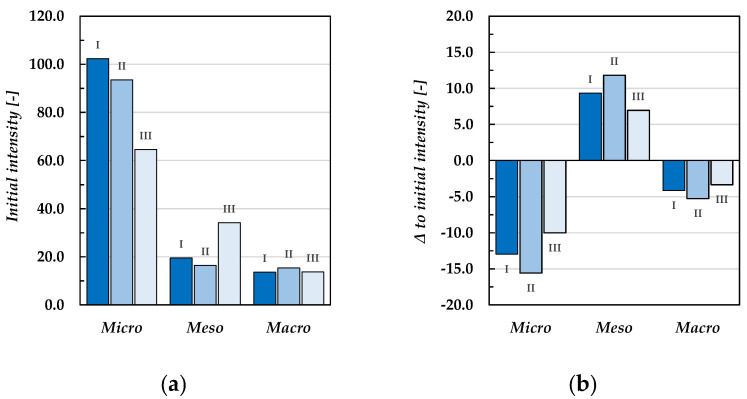
Initial moisture distribution from NMR measurements for three samples (I, II and III) before fatigue exposure (**a**) and redistributed moisture content from testing after stopping in phase II (**b**) for mortar of RH1 [22].

The NMR investigations were performed in order to gain further insight into the nano- and micro-structural effects of fatigue degradation. The results of multiexponential fitting to the averaged NMR relaxation signal are provided in Figure 5. The samples show various initial moisture distributions extending from the micro to the macro scale. The scatter between samples is greater than the repeatability of the measurements on the same sample (scatter of intensity about 2 to 4 [-]) due to specific porosity features of the individual samples. Clear trends for fatigue-induced alterations are, however, visible extending beyond such scatter and pointing towards an increase in mesoscale water inclusions, while quantities of micro- and macroscale water inclusions decrease. It may be established that fatigue exposure in Phase II alters the microstructural moisture distribution while it could not be determined whether the changes observed arise from microstructural degradation or time-dependent moisture redistribution.

## 4. Model for Water-Induced Damage

**Figure 6:**
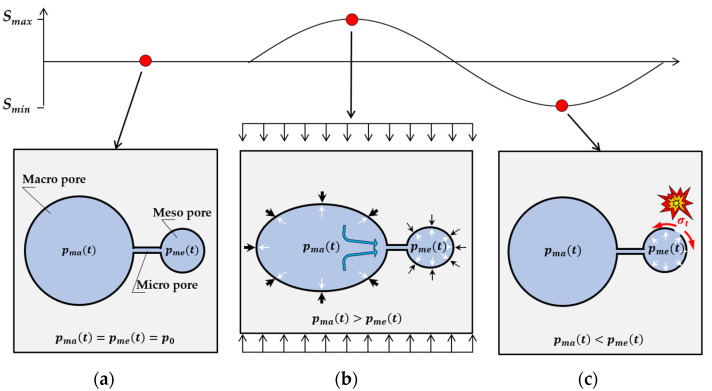
Descriptive model for water-induced damage due to cyclic loading: (**a**) first status (mean stress level); (**b**) second status (maximum stress level); (**c**) third status (minimum stress level) [22].

Based on the results of the experimental investigations, a descriptive model was derived by *Tomann* [22], which identifies the pore water pressure within the structure of cement paste as a water-induced damage mechanism. Figure 6 displays the main aspects of this model, which is described hereafter. For further details, see [22].

Water within the pore structure of hardened cement paste is located in different pore sizes, labelled macro- and mesopores that are connected via smaller micro pores. The pressure in the pore space at mean stress level is in a state of equilibrium and no pressure-related moisture relocation occurs (Figure 6a). Water is moved from the macro pores towards meso pores via the much smaller micro pores (Figure 6b) due to the increased pressure in cyclic loading towards *S_max_* and inhomogeneous volumetric deformation of the different pore types. External pressure on the pore system from loading prevents high tensile stresses at this stage. During unloading towards *S_min_*, water relocates from the meso towards the macro pore to reach an equilibrium state once again, but backflow is hindered by the small size of the micropores. A phase shift between the unloading of the external load and remaining water pressure from the previously increased mesopore moisture content arises that may exceed the typical tensile strength of the pore walls in the mesopores (Figure 6c). As a result, cracks in the pore walls do not occur at the maximum external loading, i.e., when *S_max_* is reached, but during unloading towards *S_min_*. This leads to an accelerated fatigue damage process depending on the moisture content of the sample, which goes along well with the emerging of AE hits in the unloading phase of samples with a high moisture content (see Figure 4).

## 5. Computational Model

### 5.1. Mathematical Description of Fatigue Behaviour in Poroelastic Media

Let a given domain be described for material points $x\in \Omega \in {ℛ}^{\delta }$ with a dimension δ={2,3} in the special direction, time t∈T=[0,T] and ∂Ω its surface boundary. A boundary value problem (BVP) for fluid-saturated porous media at fracture is described through a coupled three-field system. The BVP solution indicating the displacement field u(x,t), the fluid pressure field p(x,t) and the phase-field fracture d(x,t) is defined as follows:

(2) $$u:\{\begin{array}{c} \Omega \times T\to {ℛ}^{\delta }\\ \left(x,t\right)\to u\left(x,t\right) \end{array};\;p:\{\begin{array}{c} \Omega \times T\to R\\ \left(x,t\right)\to p\left(x,t\right) \end{array};\;d:\{\begin{array}{c} \Omega \times T\to \left[0,1\right]\\ \left(x,t\right)\to d\left(x,t\right) \end{array}$$

Additionally, d(x,t)=1 and d(x,t)=0 are referred to the intact and completely fractured part of the material, respectively [28]. The symmetric strain tensor under small deformation setting defines through the gradient by:

(3) $$\varepsilon =\nabla_{s}u=sym\left[\nabla u\right]:=\frac{1}{2}\left[\nabla u+\nabla u^{T}\right].$$

Additionally, a fatigue accumulation variable is introduced as

(4) $$\alpha :\;\{\begin{array}{c} \Omega \times T\to {ℛ}^{+}\\ \left(x,t\right)\to \alpha \left(x,t\right) \end{array}$$

Following Table 2, two different cases are used in our simulations: dry and wet situations. Each case includes three phases with hydrated cement paste, the clinker particle, and the dry or saturated voids according to the storage conditions. In order to formulate constitutive energy density functions for poroelasticity, the total constitutive energy was defined as follows:

(5) $$W=W_{elas}\left(\varepsilon ,d\right)+W_{fluid}\left(\varepsilon ,\theta \right)+\overset{t}{\underset{0}{\int }}f\left(\alpha \right)\frac{d}{dt}W_{frac}\left(d,\nabla d\right)$$

based on the fluid volume ratio θ. Here, total energy functional is additively decomposed into an elastic contribution $W_{elas}$, fluid flow energy $W_{fluid}$ and the fracture energy density $W_{frac}$. Hereby, f(α)>0 is a non-negative degradation function due to the fatigue load, which renders the dissipated energy for a path-dependent quantity. The elastic energy density $W_{elas}$ is formulated through the effective strain energy density $\psi_{elas}$ with a monotonically decreasing quadratic degradation function $g\left(d\right)={\left(1-d\right)}^{2}+\chi$ (see [29]).

(6) $$W_{elas}\left(\varepsilon ,p,d\right):=g\left(d\right)\;\psi_{elas}\left(\varepsilon \right)\;\mathrm{with}\;\psi_{elas}\left(\varepsilon \right):=\frac{\lambda }{2}tr^{2}\left[\varepsilon \right]+\mu tr[\varepsilon^{2}]$$

Here, λ, μ are denoted as Lamé constants. Please note that a small residual stiffness χ is introduced in the degradation function to prevent numerical instabilities. Following [30,31,32], the fluid density function subsequently takes the following quadratic form:

(7) $$W_{fluid}\left(\varepsilon ,\theta \right)=\frac{M}{2}\left[B^{2}tr^{2}\left[\varepsilon \right]-2B\theta tr\left[\varepsilon \right]+\theta^{2}\right]=\frac{M}{2}{\left(Btr\left[\varepsilon \right]-\theta \right)}^{2}$$

Here, Biot’s coefficient B  and Biot’s modulus M are used. Finally, within a regularized fracture framework, the sharp-crack surface topology denoted by C  is further regularized by the regularized crack surface functional $C_{l}$ through smeared crack surface density function given by $\gamma_{l}$ per unit volume of the solid. Additionally, the length scale parameter is denoted as l, which governs the fracture diffusivity. So, we have:

(8) $$C_{l}\left(d\right)=\int_{B}\gamma_{l}\left(d,\nabla d\right)dV\;\mathrm{with}\;\gamma_{l}=\frac{1}{2l}d^{2}+\frac{l}{2}{\left|\nabla d\right|}^{2}$$

Hence, the fracture contribution $W_{frac}\;$ of pseudo-energy density takes the following explicit form [33,34], where $\psi_{c}$ is the critical fracture density energy.

(9) $$W_{frac}\left(d,\nabla d\right)=\left[1-g\left(d\right)\right]\psi_{c}+2\psi_{c}l\gamma_{l}\left(d,\nabla d\right)$$

The critical elasticity density function $\psi_{c}$ takes the following form:

(10) $$\psi_{c}=\frac{\sigma_{c}^{2}}{2E_{cement}}$$

where the critical effective stress $\sigma_{c}$ and the cement-based Young’s modulus $E_{cement}$ have been used [30,34]. The constitutive functions and corresponding strong forms for poroelastic media are described in the following.

#### 5.1.1. Elastic Contribution

According to the classical Terzaghi theorem, the constitutive modelling corresponds to $W_{elas}$ results in the additive split of the stress tensor σ to the effective mechanical contribution and fluid part as:

(11) $$\sigma \left(\varepsilon ,p,d\right):=\frac{\partial W}{\partial \varepsilon }=\sigma_{eff}-BpI\;\mathrm{with}\;\sigma_{eff}=g\left(d\right)\left[\;\lambda tr\left[\varepsilon \right]I+2\mu \varepsilon \right]$$

Thus, the balance of linear momentum takes the following form:

(12) $$\mathrm{Div}\;\sigma \left(\varepsilon ,p,d\right)+\bar{b}=0$$

where $\bar{b}$ is the body force imposed on the domain.

#### 5.1.2. Fluid Contribution

The fluid volume flux H(ε,d,p) is described in the opposite direction of the ∇p which follows:

(13) H:=−K(ε,d)∇p

Here, the second-order permeability tensor K(ε,d) [31] is additively decomposed into a Darcy-type flow for the unfractured porous medium $K_{Darcy}$ and Poiseuille-type flow in a completely fractured material $K_{frac}$ by:

(14) $$\begin{array}{ccc} K\left(\varepsilon ,d\right) & = & K_{Darcy}+d^{\zeta }K_{frac\;}\left(\varepsilon ,d\right)\\ K_{Darcy} & = & \frac{K_{D}}{\eta_{F}}I\\ K_{frac}\left(\varepsilon ,d\right) & = & \left(\frac{\omega_{d}^{2}}{12\eta_{F}}-K_{D}\;\right)I-n⊗n \end{array}$$

where $K_{D}$ is the isotopic intrinsic permeability of the pore space, $\eta_{F}$ is the dynamic fluid viscosity and ζ is a permeability transition exponent. Additionally, the crack opening displacement $\omega_{d}$ reads:

(15) $$\omega_{d}:=〚u(x)〛.n=\left(n.\varepsilon n\right)\;h_{e}\;\mathrm{with}\;n=\frac{\nabla d}{\left|\nabla d\right|}$$

where n represents the outward unit normal to the fracture surface and $h_{e}$ is the characteristic element length. The continuity equation for the fluid mass which reflects the fluid flow PDE [31] within a hydraulic fracturing setting is:

(16) $$\dot{\theta }+\mathrm{Div}\left[H\right]=0,$$

The fluid pressure p using Coleman-Noll inequality condition reads:

(17) $$p\left(\varepsilon ,\theta \right):=\frac{\partial W}{\partial \theta }=\frac{\partial W_{fluid}}{\partial \theta }=\theta M-MB\;tr\left[\varepsilon \right]$$

So, the modified overall mass balance by condensing out the fluid volume ratio θ in (17), in terms of p, follows as

(18) $$\frac{\dot{p}}{M}+B\;tr\left[\dot{\varepsilon }\right]+\mathrm{Div}\left[H\right]=0$$

#### 5.1.3. Fatigue Contribution

Recall that the regularized fracture energy density $W_{frac}\left(d,\nabla d\right)$ is scaled by a non-negative degradation function f(α). The second axiom of thermodynamics for the fatigue response is satisfied by the non-negativity of the dissipation rate, i.e.,

(19) $$f\left(\alpha \right)\frac{d}{dt}W_{frac}\left(d,\nabla d\right)\geq 0$$

Hence, the following form was adopted according to [35,36], where k is a constant to adapt the rate of decay of the fatigue degradation function.

(20) $$f\left(\alpha \right)=\{\begin{array}{ccc} 1 & \mathrm{if} & \alpha \left(x,t\right)\leq \alpha_{c}\\ {}[1-k\log{\left(\frac{\alpha \left(x,t\right)}{\alpha_{c}}\right)]}^{2} & \mathrm{if} & \alpha \left(x,t\right)\leq \alpha_{c}10^{1/k}\\ 0 & & \mathrm{otherwise} \end{array}$$

Here, $\alpha_{c}\geq 0$ is a material threshold parameter, below which no fatigue effects are triggered. The fatigue variable α is defined as an accumulation of strain energy density $W_{elas}\left(\varepsilon ,d\right)$ during loading stages, thus:

(21) $$\alpha \left(x,t\right):=\int_{0}^{t}\dot{\vartheta }\left(x,s\right)\;\bar{H}\left(\dot{\vartheta }\left(x,s\right)\right)ds\;\mathrm{with}\;\vartheta \left(x,t\right):=W_{elas}\left(\varepsilon ,d\right)$$

Here, $\bar{H}\left(x\right)$ refers to the Heaviside function. The third PDE in the rate-dependent setting for the fatigue-induced damage yields:

(22) $$\left[d-l^{2}\Delta d\right]+\left(d-1\right)ℋ=0$$

Here, ℋ indicates the crack driving force which is derived through the maximum expression of the positive crack driving state function D(x,ε).

(23) $$ℋ\left(\varepsilon \right)=\underset{s\epsilon \left[0,t\right]}{\max}D\left(x,s\right)\geq 0\;\mathrm{with}\;D:=\;\frac{\psi_{elas}\left(\varepsilon \right)}{f\left(\alpha \right)\psi_{c}}-1_{+}$$

The Macaulay bracket is used in this equation. For an alternative description of crack irreversibility, see [29]. For recent works on the phase-field modelling of fatigue failure behaviour, the interested reader is referred to [37,38,39,40,41].

### 5.2. Poroelastic Simulation to Fatigue Load

**Figure 7:**
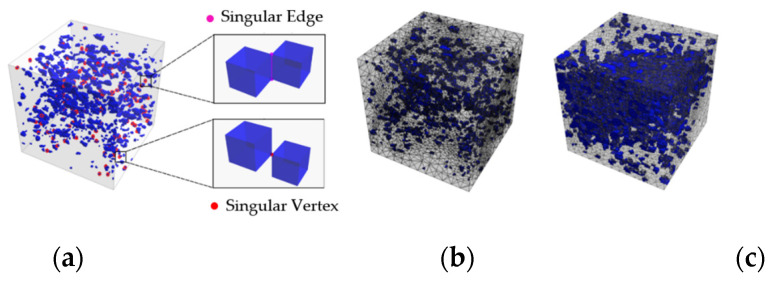
Finite element discretization: singularities inside the microstructure geometry scanned (**a**); two different high-strength concrete meshes (**b**,**c**).

The capability of the phase-field damage modelling due to the fatigue load is illustrated by a representative numerical example. The boundary value problem, and material properties, are described in previous works [28,33]. Following [30], two types of pores are used in the concrete material; see blue colour in Figure 7. Capillary pores (10 nm–100 µm) develop because of stoichiometrically unnecessary water. Additionally, Gel-pores (0.1 nm–10 nm) are initiated due to chemistry of the hydration process. The water influence on the fatigue deterioration of high-strength concrete was investigated in the following.

**Figure 8:**
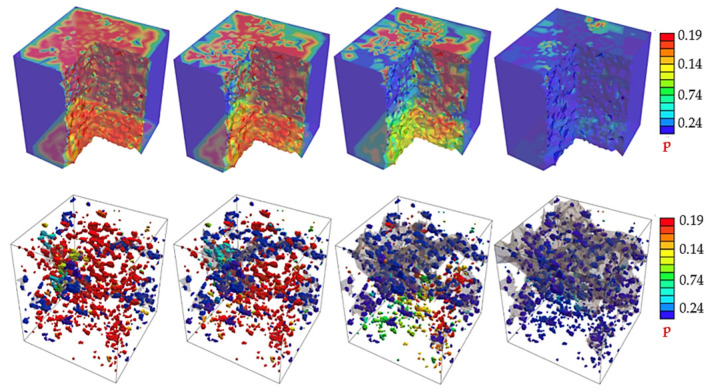
Pressure field p for the microscopic concrete specimen with different deformation states.

Next, the pressure evolution at a cut inside the high-strength concrete specimen is provided in Figure 8 (first row). The numerical solution results in the prediction of the crack phase-field d that is initiated and propagated in random directions (and so merging) inside the cement matrix until complete failure. It was noticed from the results that the crack evolution leads to pressure drops in the fracture area. The fracture evolution continues until complete failure. The pressure distribution in the pores is demonstrated in Figure 8 (second row), where red areas represent the high pressure that drops when fracture (in grey colour) starts.

**Figure 9:**
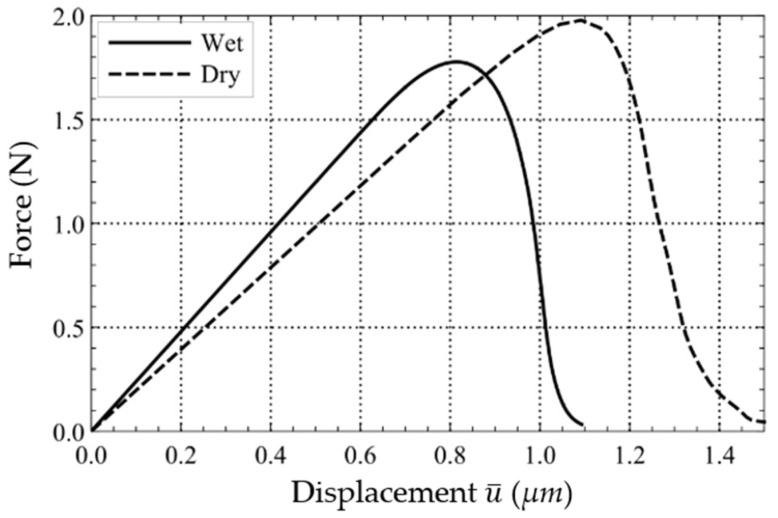
Force-displacement curves of the high-strength concrete specimen in the dry and water-saturated states.

The resulting force-displacement curves in the dry and water-saturated state are investigated in Figure 9. It can be observed that the water-saturated test specimen results in earlier damage behaviour compared to the dry test specimen.

**Figure 10:**
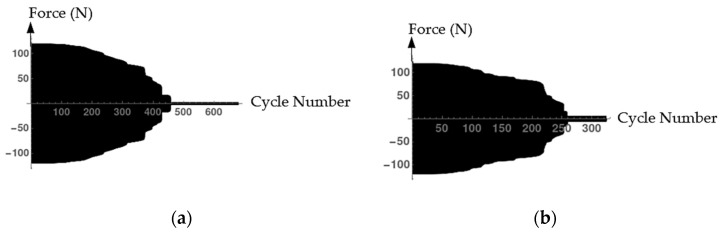
Forces versus number of cycles. (**a**) The dry case; (**b**) The wet case.

As an extension of the previous investigations, the influence of water saturation on damage under cyclical loading is considered. The number of cycles to failure versus forces for both dry and wet cases are presented in Figure 10. As expected, the force decreases with the increased number of cycles. In line with this, the fatigue resistance of high-strength concrete decreases with the increasing number of cycles to failure. The results of the numerical simulation in Figure 10 depict that the water-saturated concrete specimen is damaged earlier than the dry specimen. These results are in agreement with the experimental investigation discussed earlier.

## 6. Conclusions

The purpose of the current paper was to determine the influence of the moisture content and the wet environment on the fatigue behavior of high-strength concrete. Experimentally, the fatigue tests were conducted on high-strength concrete specimens with different moisture content. The fatigue tests were carried out at constant stress levels and load frequency throughout the entire set of investigations regarding the impact of moisture content (*S_min_* = 0.05, *S_max_* = 0.65 and *f_t_* = 1.0 Hz). The impact of load frequency on the water-induced fatigue damage was examined additionally using load frequencies of *f_t_* = 0.35–10 Hz. The numbers of cycles to failure were determined and compared with S-N curves of DNV GL [23] for wet conditions and the S-N curve of *fib* Model Code 2010 [24] for dry conditions. The developments of stiffness and AE hits were analysed in addition as damage indicators and discussed along with results of microstructural investigations to provide insights into the moisture-dependent degradation mechanisms. On the numerical side, a simulation model was developed by means of phase-field modelling, which allows simulations of the fatigue damage behaviour of dry and water-saturated high-strength concretes. The main results of the experimental and numerical investigations can be summarized as follows:

- The number of cycles to failure showed a significant reduction with increasing moisture contents in the concrete. The largest difference in the numbers of cycles to failure of Δ*log_10_ N_f_* = 2.5 was found between water-saturated and dried concrete specimens.
- The moisture content in the concrete—and not the water as an environment—was identified as being primarily responsible for accelerated fatigue deterioration of concrete.
- The results for the development of stiffness showed an increased stiffness degradation per load cycle with increasing moisture content. The results indicated additionally the presence of a certain moisture content, beyond which different or additional mechanisms of fatigue damage arise.
- The water-induced fatigue damage was distinguished from conventional fatigue damage based on the occurrence of the AE hits. Compared to specimens stored in a climate chamber, the point of occurrence of AE hits of water-saturated concrete specimens shifts from a maximum to minimum stress level. This difference in the results confirms that additional damage mechanisms are acting with increasing moisture content in concrete, which lead to an accelerated degradation process due to fatigue loading.
- Investigations with respect to the influence of the load frequency show a significant reduction of fatigue resistance and increasing stiffness degradation per load cycle in phase II with decreasing load frequency.
- Considering the moisture distribution results from NMR testing, moisture redistribution and degradation patterns were visible in the size range predicted by *Tomann* [22] for the wet-fatigue mechanism, confirming this hypothesis.
- The numerical results confirm that the fatigue effect for water-saturated concretes leads to a reduction in the damage resistance compared to dry concretes. The numerical prediction coincides well with the results from experiments.

In spite of these findings and advances, a detailed understanding of water-induced damage mechanisms is still lacking. For this purpose, further microstructural investigations regarding porosity, pore size distribution and nanoscale moisture content are to be conducted in further stages of the research project. The impact of loading frequency and further parameter such as stress level on water-induced fatigue damage are investigated and analysed by means of AE analysis. The modelling of damage mechanisms is validated from the results of microstructural investigations.

## Figures and Tables

**Table 1:** Composition of the concrete RH1.

Components	Content (kg/m^3^)
CEM I 52.5 R-HS/NA (Holcim Sulfo, Lägerdorf, Germany)	500
Quartz sand (0/0.5 mm) (Quarzwerke H33, Haltern, Germany)	75
Sand (0/2 mm) (Tündern, Germany)	850
Basalt (2/5 mm) (Ölberg, Germany)	350
Basalt (5/8 mm) (Ölberg, Germany)	570
Superplasticiser (BASF MasterGlenium^®^ ACE 460)	5.00
Stabiliser (BASF MasterMatrix^®^ SDC 100)	2.85
Water	176

**Table 2:** Definition of the storage and test conditions.

Acronym	Storage Conditions	Test Conditions
**D**	Dried	Sealed
**C**	Climate chamber	Sealed
**M**	Sealed	Sealed
**WS**	Water storage	Sealed
**WST**	Water storage	Underwater

**Table 3:** The number of fatigue tests.

Concrete	Load Frequency *f_t_*	Number of Fatigue Tests per Storage Condition				
		D	C	M	WS	WST
RH1-B	0.35 Hz	-	-	-	-	3
RH1-B/HPC-1	1 Hz	1	2	4	4	3/3
RH1-B	5.0 Hz	-	-	-	-	3
RH1-B	10.0 Hz	-	-	-	-	3

## Data Availability

Data available on request.

## Appendix A

**Table A1:** Material properties of RH1 and HPC-1 [22].

Storage Conditions	Moisture Content [mass-%]		*f_cm,_*_150_ [MPa]	
	RH1	HPC-1	RH1	HPC-1
**D**	~0	-	108	-
**C**	3.5	-	97	-
**M**	4.3	-	107	-
**WS/WST**	5.0	4.0	110	107

**Table A2:** The numbers of cycles to failure depending on the storage conditions for RH1 [22].

Storage Conditions	*f_t_*	*S_max_*	*S_min_*	*N_f_*	log *N_f_*	Mean log *N_f_*
**D**	1	0.65	0.05	5,000,000	6.70	6.70
**C**	1	0.65	0.05	199,827	5.30	5.32
	1	0.65	0.05	217,093	5.34	
**M**	1	0.65	0.05	41,258	4.62	4.51
	1	0.65	0.05	27,030	4.43	
	1	0.65	0.05	36,688	4.56	
	1	0.65	0.05	27,907	4.45	
**WS**	1	0.65	0.05	16,581	4.22	4.27
	1	0.65	0.05	16,534	4.22	
	1	0.65	0.05	28,963	4.46	
	1	0.65	0.05	14,460	4.16	
**WST**	1	0.65	0.05	16,331	4.21	4.20
	1	0.65	0.05	10,485	4.02	
	1	0.65	0.05	22,710	4.36	

**Table A3:** The number of cycles to failure depending on the load frequency for HPC-1 [22].

Storage Conditions	*f_t_*	*S_max_*	*S_min_*	*N_f_*	log *N_f_*	Mean log *Nf*
**WST**	10	0.65	0.05	24,271	4.39	4.42
	10	0.65	0.05	31,153	4.49	
	10	0.65	0.05	23,499	4.37	
**WST**	5	0.65	0.05	16,208	4.21	4.22
	5	0.65	0.05	22,887	4.36	
	5	0.65	0.05	12,329	4.09	
**WST**	1	0.65	0.05	13,931	4.14	4.05
	1	0.65	0.05	9944	4.00	
	1	0.65	0.05	10,396	4.02	
**WST**	0.35	0.65	0.05	3880	3.59	3.69
	0.35	0.65	0.05	4494	3.65	
	0.35	0.65	0.05	6779	3.83	

## References

[1] Muguruma H., Watanabe F. On the Low-cycle Compressive Fatigue Behaviour of Concrete under Submerged Condition. Proceedings of the 27th Japan Congress on Materials Research.

[2] Hohberg R. (2004). Zum Ermüdungsverhalten von Beton. [About the Fatigue Behaviour of Concrete]. Ph.D. Thesis.

[3] Sørensen E.V., Westhof L., Yde E., Serednicki A. Fatigue Life of High Performance Grout for Wind Turbine Grouted Connection in Wet or Dry Environment. Paper Presented at EWEA OFFSHORE 2011.

[4] Oneschkow N., Hümme J., Lohaus L. (2020). Compressive fatigue behaviour of high-strength concrete in a dry and wet environment. Constr. Build. Mater..

[5] Hümme J. (2018). Ermüdungsverhalten von hochfestem Beton unter Wasser. Hannover. [Fatigue Behaviour of High-strength Concrete under Water]. Ph.D. Thesis.

[6] Hümme J., Lohaus L. Fatigue Behaviour of High-strength Grout in Dry and Wet Environment. Proceedings of the International Wind Engineering Conference IWEC.

[7] Nygard K., Petković G., Rosseland S., Stemland H. (1992). The Influence of Moisture Conditions on the Fatigue Strength of Concrete. High Strength Concrete.

[8] Oneschkow N. (2014). Analyse des Ermüdungsverhaltens von Beton anhand der Dehnungsentwicklung. [Analysis of the Fatigue Behaviour of Concrete with Respect to the Development of Strain]. Ph.D. Thesis.

[9] Pesch A. (1997). Ein Beitrag zum zeitabhängigen Verhalten von hochfestem Beton und hochfestem Mörtel [A Contribution to the Time-dependent Behaviour of High-strength Concrete and High-strength Mortar]. Ph.D. Thesis.

[10] Brosge S. (2001). Beitrag zur Ermüdungsfestigkeit von Hochfestem Beton [Contribution to the Fatigue Strength of High-Strength Concrete]. Ph.D. Thesis.

[11] Peterson W.S. (1980). Fatigue of reinforced concrete in sea water. Spec. Publ..

[12] Aldakheel F., Tomann C., Lohaus L., Wriggers P. (2019). Water-induced failure mechanics for concrete. PAMM.

[13] Tomann C., Oneschkow N. (2019). Influence of moisture content in the microstructure on the fatigue deterioration of high-strength concrete. Struct. Concr..

[14] Basaldella M., Oneschkow N., Ludger L. (2021). Influence of the specimen production and preparation on the compressive strength and the fatigue resistance of HPC and UHPC. Mater. Struct..

[15] German Institute for Standardization (2019). DIN EN 12390-3:2019-10. Testing Hardened Concrete—Part 3: Compressive Strength of Test Specimens.

[16] German Institute for Standardization (2018). DIN EN ISO 7500-1:2018-06. Metallic Materials—Calibration and Verification of Static Uniaxial Testing Machines—Part 1: Tension/Compression Testing Machines—Calibration and Verification of the Force-Measuring System.

[17] Tomann C., Lohaus L., Aldakheel F., Wriggers P. Influence of Water-induced Damage Mechanisms on the Fatigue Deterioration of High-strength Concrete. Proceedings of the fib Symposium 2019: Concrete—Innovations in Materials, Design and Structures.

[18] Hardy E.H. (2012). NMR Methods for the Investigation of Structure and Transport.

[19] Wyrzykowski M., McDonald P.J., Scrivener K.L., Lura P. (2017). Water Redistribution within the Microstructure of Cementitious Materials due to Temperature Changes Studied with 1H NMR. J. Phys. Chem. C.

[20] Radel B., Hardy E.H., Djuric Z., Mahlbacher M., Haist M., Müller H.S. (2019). Regularized inversion of the Laplace transform for series of experiments. Magn. Reson. Chem..

[21] Valori A., McDonald P.J., Scrivener K.L. (2021). The morphology of C–S–H: Lessons from 1H nuclear magnetic resonance relaxometry. Cement Concr. Res..

[22] Tomann C. (2014). Wasserinduzierte Ermüdungsschädigung von Beton. [Water-induced Fatigue Damage of Concrete]. Ph.D. Thesis.

[23] DNVGL Offshore Standards (2018). DNV-ST-C502 Offshore Concrete Structures.

[24] Fédération International du Béton (2013). Fib Model Code for Concrete Structures 2010.

[25] Markert M., Veit B., Garrecht H. Temperature and Humidity Induced Damage Processes in Concrete Due to Pure Compressive Fatigue Loading. Proceedings of the fib Symposium 2019: Concrete—Innovations in Materials, Design and Structures.

[26] Markert M., Veit B., Garrecht H. (2019). Influence of Concrete Humidity on the Temperature Development under Fatigue Compressive Loading. Proceedings of the IOP Conference Series: Materials Science and Engineering.

[27] Oneschkow N. (2016). Fatigue behaviour of high-strength concrete with respect to strain and stiffness. Int. J. Fatigue.

[28] Noii N., Fan M., Wick T., Jin Y. (2021). A quasi-monolithic phase-field description for orthotropic anisotropic fracture with adaptive mesh refinement and primal–dual active set method. Eng. Fract. Mech..

[29] Noii N., Khodadadian A., Ulloa J., Aldakheel F., Wick T., Francois S., Wriggers P. (2021). Bayesian inversion for unified ductile phase-field fracture. Comput. Mech..

[30] Aldakheel F. (2020). A microscale model for concrete failure in poro-elasto-plastic media. Theor. Appl. Fract. Mech..

[31] Noii N., Khodadadian A., Wick T. (2021). Bayesian inversion for anisotropic hydraulic phase-field fracture. Comput. Method Appl. Mech. Eng..

[32] Aldakheel F., Noii N., Wick T., Wriggers P. (2020). A global–local approach for hydraulic phase-field fracture in poroelastic media. Comput. Math. Appl..

[33] Aldakheel F., Noii N., Wick T., Allix O., Wriggers P. (2021). Multilevel global–local techniques for adaptive ductile phase-field fracture. Comput Method Appl. Mech. Eng..

[34] Wriggers P., Aldakheel F., Lohaus L., Haist M. (2020). Water-induced damage mechanisms of cyclically loaded high-performance concretes. Bauingenieur.

[35] Ulloa J., Wambacq J., Alessi R., Degrande G., François S. (2020). Phase-field modeling of fatigue coupled to cyclic plasticity in an energetic formulation. Comput. Method Appl. Mech. Eng..

[36] Carrara P., Ambati M., Alessi R., De Lorenzis L. (2019). A framework to model the fatigue behavior of brittle materials based on a variational phase-field approach. Comput. Method Appl. Mech. Eng..

[37] Storm J., Pise M., Brands D., Schröder J., Kaliske M. (2020). A comparative study of micro-mechanical models for fiber pullout behavior of reinforced high performance concrete. Eng. Fract. Mech..

[38] Pise M., Brands D., Sarhil M., Schröder J., Zingoni A. (2019). Numerical Calibration of Elasto-plastic Phase-field Modeling of Fracture for Experimental Pullout Tests of Single Steel Fibers Embedded in High-performance Concrete. Proceedings of the SEMC2019: The Seventh International Conference on Structural Engineering, Mechanics and Computation.

[39] Schreiber C., Kuhn C., Müller R., Zohdi T.I. (2020). A phase field modeling approach of cyclic fatigue crack growth. Int. J. Fract..

[40] Seiler M., Keller S., Kashaev N., Klusemann B., Kästner M. (2021). Phase-field modelling for fatigue crack growth under laser shock peening-induced residual stresses. Arch. Appl. Mech..

[41] Gebuhr G., Pise M., Sarhil M., Anders S., Brands D., Schröder J., Zingoni A. (2019). Deterioration Development of Steel Fibre Reinforced High performance Concrete in Low-cycle Fatigue. Proceedings of the SEMC2019: The Seventh International Conference on Structural Engineering, Mechanics and Computation.

